# Contact Behavior of Phenyl Silicone Rubber Material Under the Condition of High-Altitude Environment

**DOI:** 10.3390/ma19194160

**Published:** 2026-09-29

**Authors:** Yipan Gao, Di Chen, Haiqiang Shi, Xuedong Xu, Li Qi, Jian Wu

**Affiliations:** 1 State Key Laboratory of Environmental Adaptability for Industrial Products, China National Electric Apparatus Research Institute Co., Ltd., Guangzhou 510663, Chinaqil@cei1958.com (L.Q.); 2 Center for Rubber Composite Materials and Structures, Harbin Institute of Technology, Weihai 264209, China; 25s130173@stu.hit.edu.cn (D.C.);; 3 Tibet SINOMACH Plateau Electromechanical Equipment Research Co., Ltd., Lhasa 850099, China

**Keywords:** phenyl silicone rubber, high-altitude environment, mechanical properties, aging mechanism

## Abstract

Phenyl silicone rubber is widely used in aircraft seals for its high- and low-temperature tolerance and chemical stability. However, intense UV radiation and elevated ozone at high altitudes accelerate aging, threatening seal reliability. The accelerated aging tests of phenyl silicone rubber seals have been carried out under UV and ozone to examine their mechanical, microstructural, and chemical evolution. Then, a finite element contact model of phenyl silicone rubber seals was developed considering the influence of UV and ozone aging. Both conditions increased hardness and elastic modulus but reduced tensile strength and elongation at break, with UV degradation markedly more severe. UV exposure caused a 29% maximum loss in elongation (compared to 16% under ozone) and a hardness rise of 2.30 HA, roughly 1.7 times that of ozone aging (1.35 HA). The contact pressure increases by approximately 15.2% and 10.7%. These findings provide a theoretical basis for performance evaluation and service-life prediction of these seals in high-altitude environments.

## 1. Introduction

High-altitude environments are characterized by low atmospheric pressure, low temperatures, large temperature fluctuations, intense solar radiation, and high concentrations of ozone [1,2], which have a profound impact on the aging behavior of polymer materials [3,4,5,6]. With an average elevation of more than 4000 m, the Qinghai–Tibet Plateau experiences 30% to 40% more solar UV radiation than plain areas at the same latitude. Additionally, ozone concentrations are much higher there than in places at medium and low elevations [7,8,9,10]. In plateau environments, rubber seals face conventional thermal-oxidative aging along with intense UV radiation and high ozone levels, rendering their aging and failure mechanisms more complex than in standard settings; thus, investigating how these factors affect the seals’ aging and mechanical properties is of significant engineering importance for the safe operation of aerospace and ground facilities in such regions.

Silicone rubber is characterized by its excellent resistance to high and low temperatures, superior chemical stability, and good elastic recovery [11,12,13], It has been widely used in aerospace sealing systems, electrical insulation, and building sealing, among other fields [14,15,16]. In particular, phenyl silicone rubber further enhances the material’s low-temperature resistance and radiation resistance by introducing phenyl side groups into the polysiloxane backbone [17,18,19,20]. Phenyl silicone rubber has become one of the ideal sealing materials for high-altitude environments. However, under long-term service, the combined effects of characteristic high-altitude factors—intense UV radiation and high ozone concentrations—inevitably trigger irreversible aging, including chain scission and cross-linking, which degrades mechanical properties and sealing performance, posing a serious threat to equipment operational reliability and safety [21,22,23].

Scholars both in China and abroad have conducted extensive research on the aging behavior of phenyl silicone rubber materials. Wang et al. demonstrated that CeO_2_ and graphene synergistically enhance the thermal-oxidative aging resistance of phenyl silicone rubber composites [24]; Quan et al. also examined the influence of thermal-oxidative aging on the tribological properties of phenyl silicone rubber and revealed an intrinsic correlation between aging degree and friction and wear behavior [25]. Han et al. investigated the thermal-oxidative aging of high-performance damping phenyl silicone rubber, showing that modification strategies enhance its mechanical properties and thermal-oxidative stability [26]. However, research on the aging and mechanical behavior of phenyl silicone rubber under high-altitude conditions (high ozone, intense UV) remains limited. Most existing work addresses general-purpose and ordinary silicone rubbers, leaving its degradation patterns, microstructural evolution, and aging mechanisms insufficiently understood, which hinders seal design and service-life assessment in such environments. Based on the above analysis, this study focuses on phenyl silicone rubber used in aerospace seals. Accelerated aging tests under ozone and ultraviolet (UV) environments were conducted to systematically investigate the effects of these two typical high-altitude environmental factors on the mechanical properties, compression set behavior, surface microstructure, and molecular chemical structure of phenyl silicone rubber. The resulting aging-related material parameters were used to calibrate a finite element contact model of the seal, enabling prediction of contact pressure evolution under different aging states and temperature conditions. By integrating macroscopic performance testing with microstructural characterization, this study clarifies the aging mechanism of phenyl silicone rubber in high-altitude environments and provides a theoretical basis and experimental support for material selection, performance evaluation, and service life prediction of phenyl silicone rubber seal structures in such environments.

## 2. Materials and Methods

### 2.1. Preparation of Material Specimens

**Figure 1:**
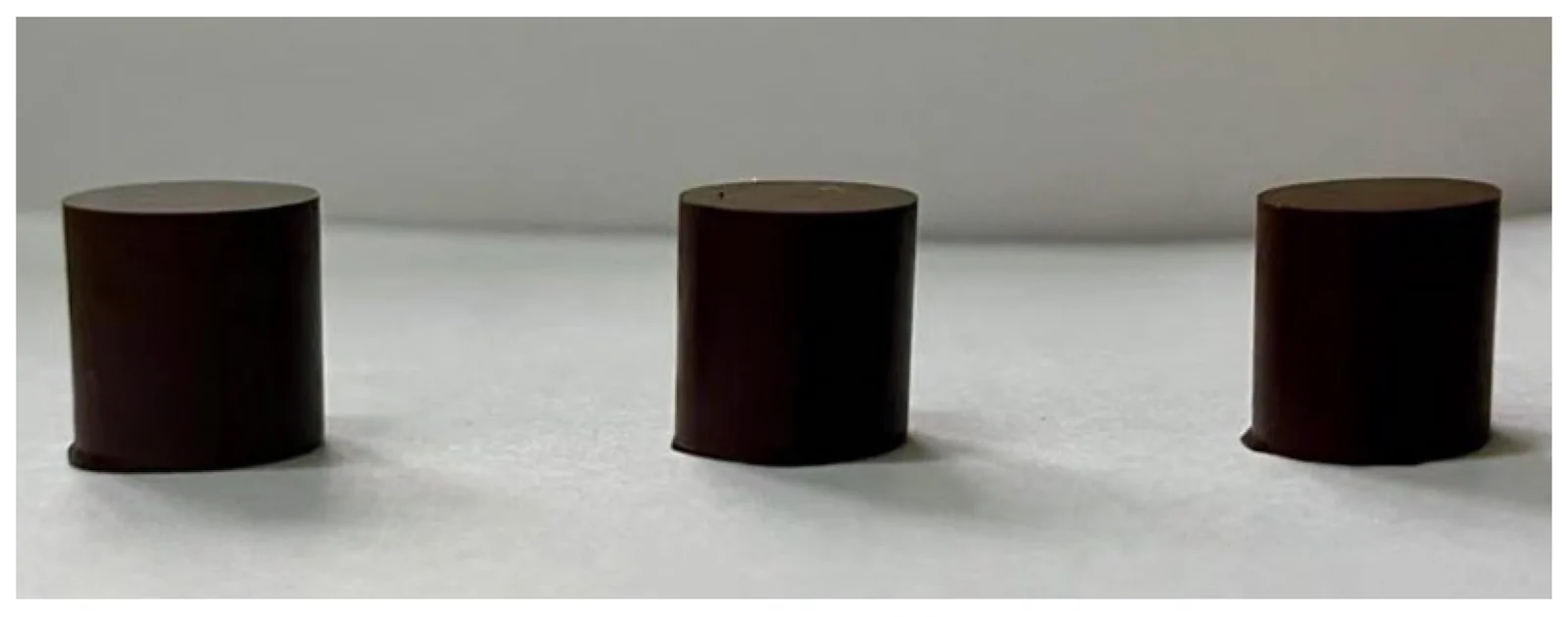
Cylindrical phenyl silicone rubber test specimens.

The phenyl silicone rubber used in this study was supplied by the Beijing Aeronautical Materials Research Institute of China Aero Engine Corporation and prepared in accordance with GB/T 2941-2025 (identical to ISO 23529:2016) [27]. After vulcanization, it was die-cut into Type 2 dumbbell specimens for uniaxial tensile testing and cylindrical specimens (Φ10.0 ± 0.2 mm in diameter and 10.0 ± 0.2 mm in height), as shown in Figure 1.

### 2.2. Accelerated Aging

The ozone aging test was conducted using an EKT-2001OZ(L) chamber (Yezhong Technology Co., Ltd., Taiwan, China) in accordance with GB/T 7762-2014 (modified from ISO 1431-1:2004), with the chamber set to 40 °C and an ozone concentration of 50 pphm to simulate high-altitude ozone conditions [28]. Ozone primarily attacks unsaturated double bonds in rubber chains at the material surface. Since this attack can cause cracking rapidly, the aging period was set to 7 days. Three specimens were prepared per aging period and collected according to the schedule in Table 1.

The UV aging test was conducted using a TZW-150UVA chamber (Wuxi Hailis Environmental Testing Equipment Co., Ltd.) equipped with a UV-A lamp. Its emission covers the UV-A band (320–400 nm) with a peak at 340 nm, and the relative spectral irradiance at the peak is typically 0.68 W/m^2^, in accordance with GB/T 16585-1996 and GB/T 14522-2008, at a black standard temperature (BST) of 80 °C and a UV irradiance of 60 W/m^2^ over the 320–400 nm range to simulate high-altitude solar UV radiation [29]. Ultraviolet light can directly break the chemical bonds in nearly all polymeric materials, leading to overall degradation, powdering, and a decline in performance [30]. This is a process that progresses from the surface inward and takes a longer time to become apparent; therefore, the maximum aging period is 56 days. Three test specimens are set for each aging period, and specimens are retrieved according to the schedule specified in Table 2.

### 2.3. Characterization

#### 2.3.1. Mechanical Property Testing

Hardness was measured using an LX-A Shore A durometer (model LX-A) in accordance with GB/T 39693.5-2025 (identical to ISO 48-5:2018). Each specimen was placed on a level surface, the durometer was pressed flush against it, and the reading was taken within 1 s; five readings per specimen were recorded, and the median was used.

Uniaxial tensile testing was performed in accordance with GB/T 528-2009 (equivalent to ISO 37:2005) using a UH4204GD universal testing machine (Shanghai Hengyu Instrument Co., Ltd., Shanghai, China) equipped with an environmental chamber and a video extensometer. Type 2 dumbbell specimens were tested at −70, −55, −25, 0, and 25 °C. Before testing, the specimens were held at the target temperature for 10 min. The crosshead speed was 100 mm/min. Three specimens were tested for each condition, and the average value was used.

#### 2.3.2. Compression Set

The compression set was determined in accordance with GB/T 1683-2018, which is technically equivalent to ISO 815. Cylindrical specimens were placed between two flat plates and compressed to 75% of their original height using spacers, corresponding to a constant compression ratio of 25%. The compressed specimens were then subjected to the same ozone aging and UV aging conditions as described in Section 2.2. At predetermined aging intervals, the specimens were removed from the aging chamber, released from the compression fixture, and allowed to recover at standard laboratory temperature for 30 min. The compression set *P* was calculated as:

(1) $$P\;=\;\frac{h_{0}-h_{1}}{h_{0}-h_{2}}\times 100\%$$

where

h_0_—Initial height of the specimen (mm);
h_1_—Height of the specimen after recovery following the test (mm);
h_2_—Height of the limiter (mm).

#### 2.3.3. Surface Topography Analysis

The microstructure was observed using a Zeiss MERLIN Compact field-emission scanning electron microscope. Prior to observation, the rubber samples were thoroughly dried, mounted with conductive adhesive, and coated with a 5–10 nm conductive layer of gold or platinum, and care was taken to ensure they were non-magnetic.

#### 2.3.4. Infrared Spectroscopy

The chemical structure was characterized using a Bruker Tensor II FTIR spectrometer (Germany) with a spectral range of 4000–400 cm^−1^, a resolution of 4 cm^−1^, and 32 scans; spectra were recorded as absorbance. Samples were analyzed by matching the absorption peaks to characteristic wavenumber ranges of molecular structures and chemical bonds.

### 2.4. Finite Element Model of the Seal

#### 2.4.1. Boundary and Load Conditions

**Figure 2:**
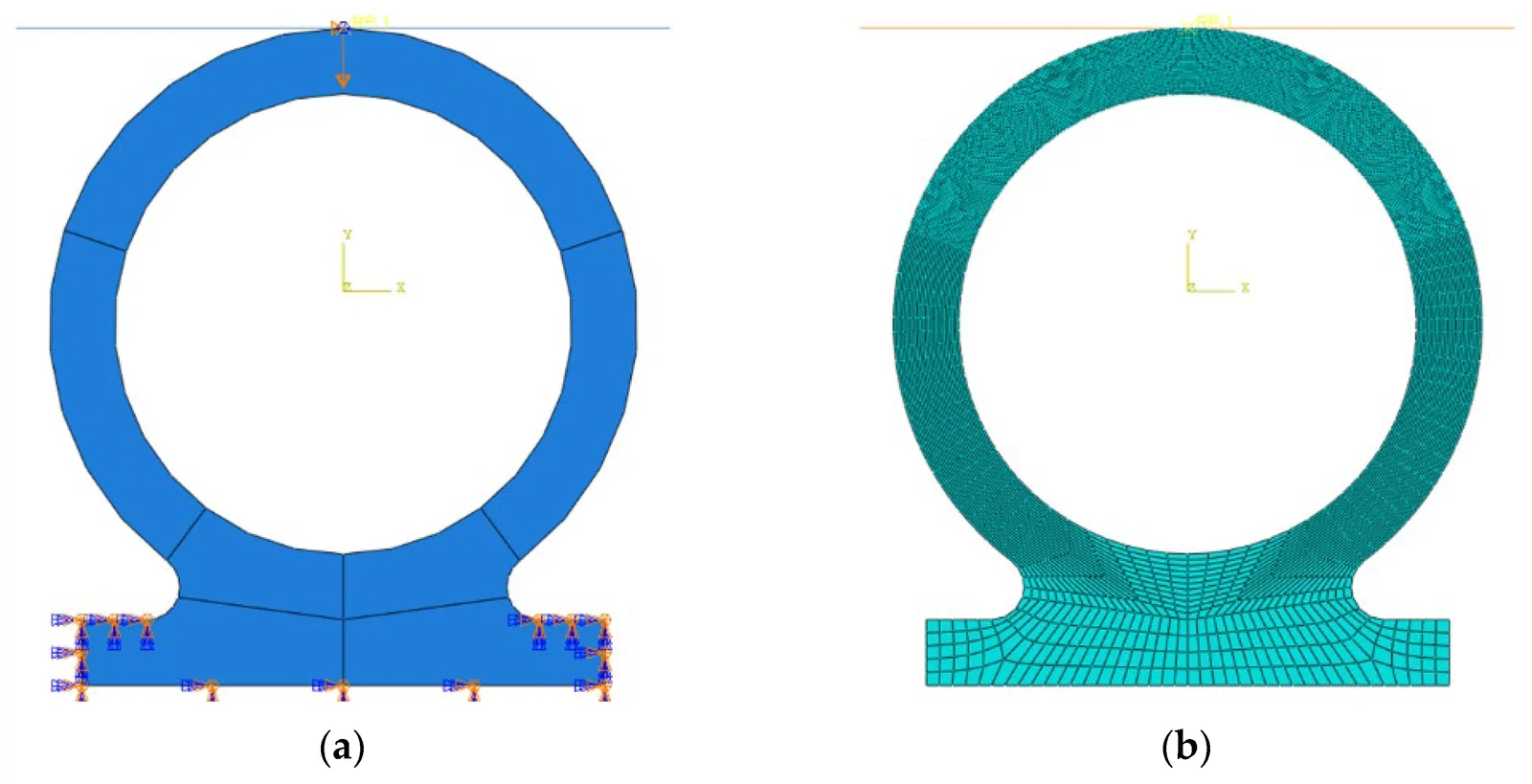
Boundary conditions and mesh generation for the omega-type seal. (**a**) Boundary and load conditions. (**b**) Mesh generation [31].

A two-dimensional finite element model of an Omega-type seal was developed in Abaqus (v2022), comprising a rigid pressure plate and a deformable rubber seal; the much stiffer plate was modeled as a rigid body, while the seal was assigned a Poisson’s ratio of 0.495 and material parameters calibrated from aging data. The seal base was fixed, and a controlled downward displacement of the pressure plate applied compression to the seal until the maximum displacement of 8 mm was reached (Figure 2a).

#### 2.4.2. Mesh Generation

After modeling, the seal structure was meshed in the Abaqus Mesh module. To balance accuracy and efficiency, a global mesh size of 0.5 mm was used, with a finer 0.1 mm mesh at the contact interface between the metal shaft and the seal. The mesh employed CPE4RH elements generated using the neutral-axis algorithm, yielding 8762 nodes and 9138 elements (Figure 2b).

## 3. Results and Discussion

### 3.1. Mechanical Property Changes of Phenyl Silicone Rubber

#### 3.1.1. Patterns of Hardness Variation

**Figure 3:**
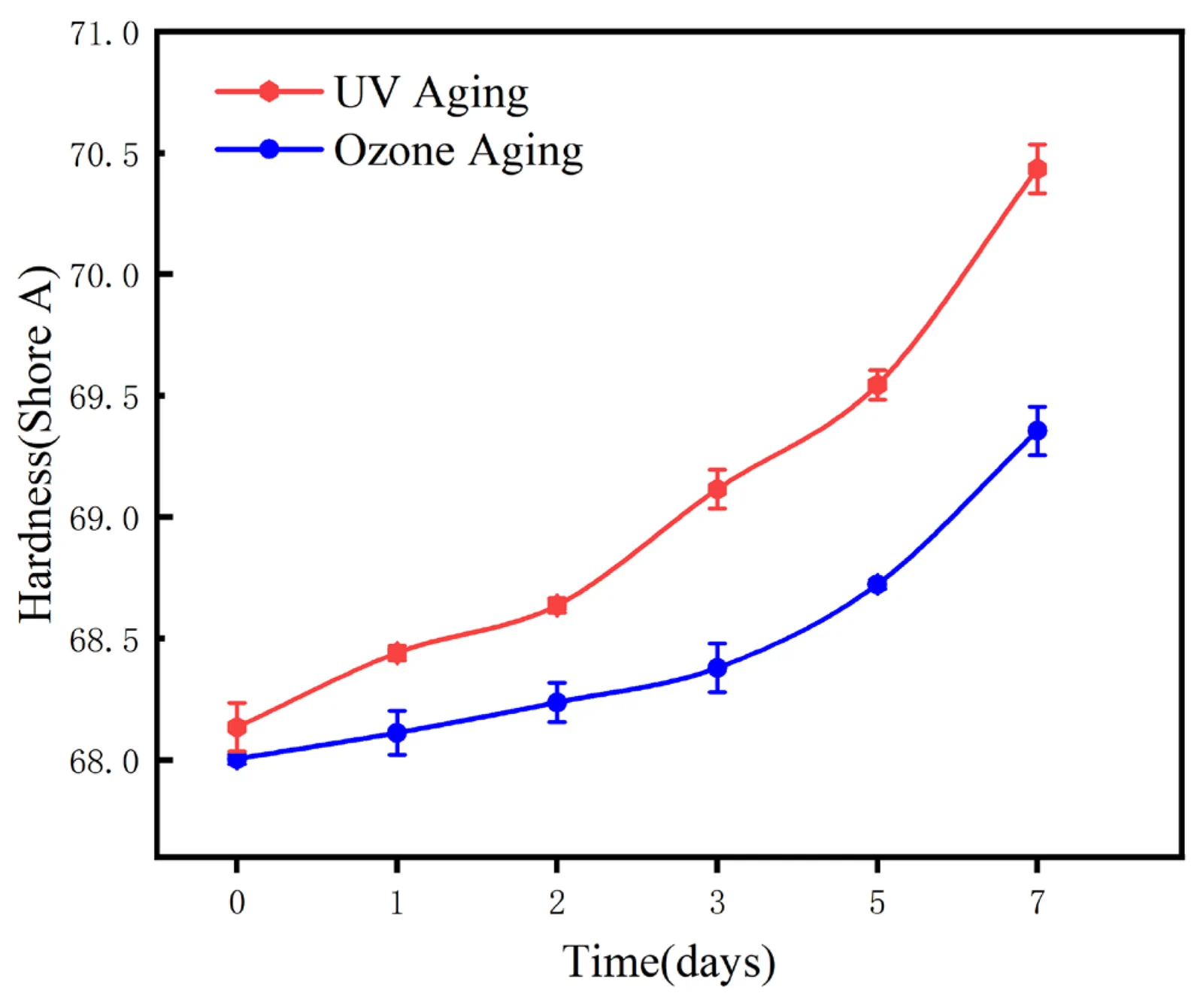
Patterns of hardness changes under different aging conditions.

As shown in Figure 3, Shore A hardness increased in both aging environments, but to different degrees. After 7 days, ozone aging raised hardness from 68.01 HA to 69.36 HA (+1.35 HA), whereas UV aging increased it to 70.43 HA (+2.30 HA)—approximately 1.7 times greater. This hardness increase reflects further densification of the cross-linked network, restricting molecular mobility and enhancing indentation resistance. The difference stems largely from the depth of attack: ozone primarily targets surface unsaturated bonds, inducing localized oxidative cross-linking, and because ozone diffusion into the matrix is limited, reactions are confined to the surface layer, yielding a relatively moderate hardness rise [32,33,34]. By contrast, UV radiation, with its higher photon energy, penetrates deeper and directly breaks internal molecular bonds, triggering more intense photo-oxidative cross-linking and chain rearrangement throughout the material, which explains the greater hardness increase [35,36,37]. In summary, UV radiation increased the hardness of phenyl silicone rubber more significantly than ozone, underscoring that light radiation strongly promotes restructuring of the bulk cross-linked network. While the hardness gain reflects enhanced rigidity and deformation resistance, it also signals a risk of elasticity loss from excessive cross-linking, potentially compromising the flexibility and compensation capacity of seals during long-term service.

#### 3.1.2. Patterns of Change in Tensile Strength

**Figure 4:**
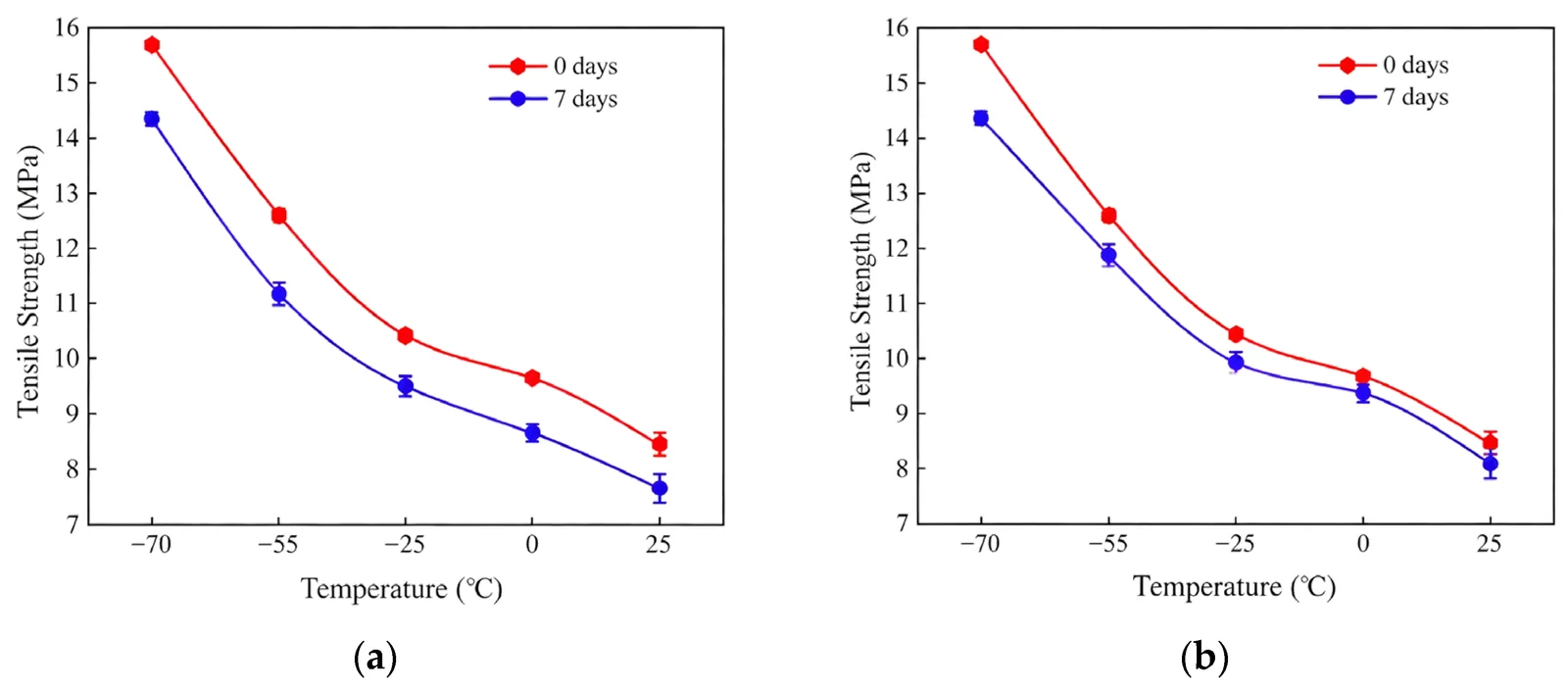
Tensile strength trends under different aging conditions. (**a**) Tensile strength change patterns under ozone aging. (**b**) Tensile strength change patterns under UV aging.

As shown in Figure 4, the tensile strength of phenyl silicone rubber declined markedly with increasing temperature in both aging environments, though the extent of degradation varied. After 7 days, ozone aging reduced the strength from 14.33 MPa at −70 °C to 7.65 MPa at 25 °C (a decrease of 6.68 MPa), while UV aging reduced it from 14.35 to 8.09 MPa (a decrease of 6.26 MPa). Compared to unaged specimens, the strength loss across the measured temperatures ranged from 0.8 to 1.4 MPa for ozone-aged samples and from 0.3 to 1.3 MPa for UV-aged samples.

The temperature dependence of tensile strength stems from changes in molecular chain mobility: at −70 °C, segmental motion is severely restricted near the glassy–hyperelastic transition, so the rigid crosslinked network bears the load and strength is high; as the temperature rises to 25 °C, segmental motion increases, chains slip and rearrange, and load-bearing capacity decreases. Aging weakens tensile strength by altering the crosslinked network—both ozone and UV exposure cause oxidative chain scission, which reduces effective load-bearing segments, and excessive crosslinking, which restricts segment realignment, intensifies stress concentration, and promotes crack initiation and propagation. Ozone attack is largely confined to the surface, so the resulting crosslink-density increase and surface microcracks more directly compromise the load-bearing cross-section, leading to greater strength loss. UV radiation penetrates more deeply but induces more uniform crosslinking that delays weak-zone formation, so the strength loss is comparatively smaller.

#### 3.1.3. Patterns in the Elongation at Break

**Figure 5:**
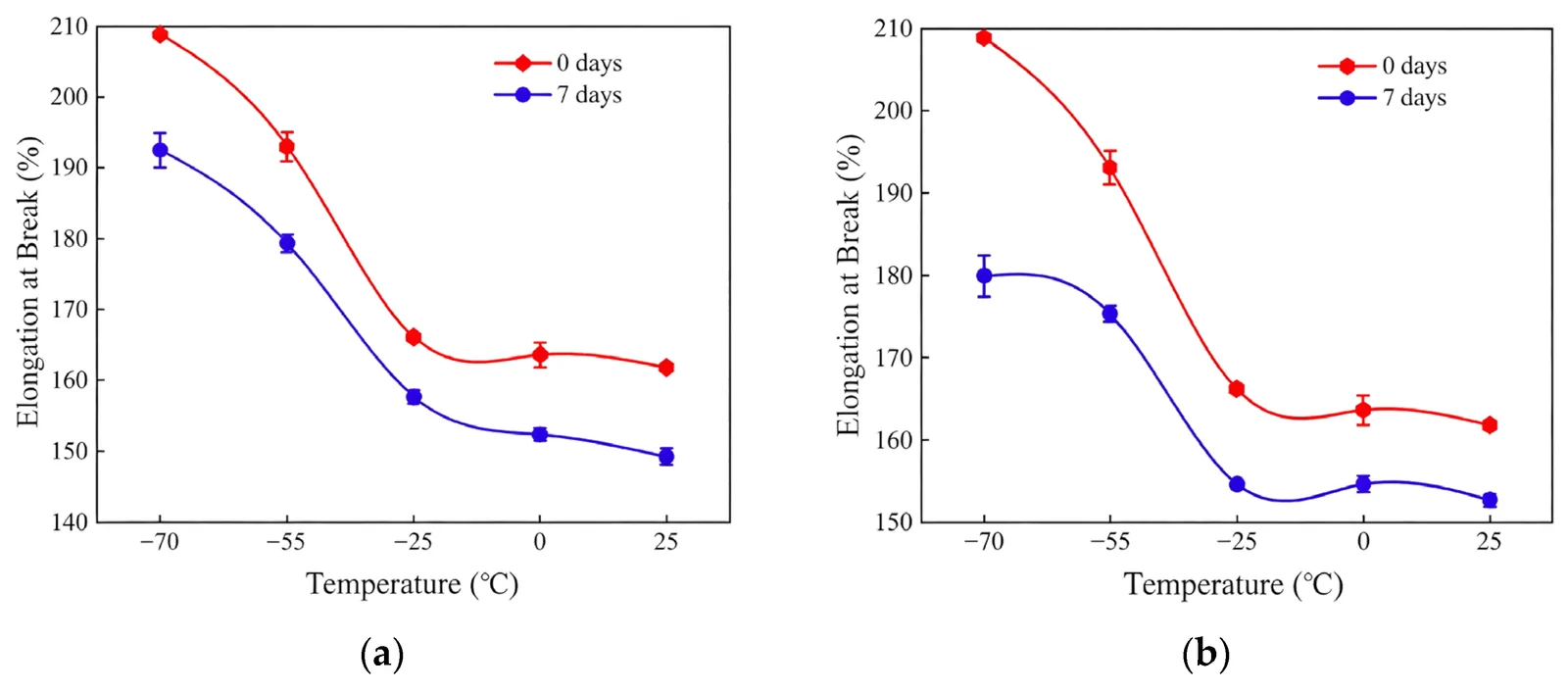
Elongation at break trends under different aging conditions. (**a**) Elongation at break change patterns under ozone aging. (**b**) Elongation at break change patterns under UV aging.

As shown in Figure 5, elongation at break decreased continuously with increasing temperature in both aging environments, but UV aging caused a far greater loss of ductility. After 7 days, ozone aging reduced elongation from 193% (−70 °C) to 149% (25 °C), representing losses of 9–16% relative to unaged specimens, while UV aging reduced it from 179% to 152%, with losses of 9–29%. The much higher maximum loss (29% vs. 16%) underscores the more severe detrimental effect of UV radiation on ductility.

The two aging environments reduce elongation at break through fundamentally different mechanisms. Ozone aging is surface-dominated: it attacks surface unsaturated bonds, sharply raising surface crosslink density and forming a rigid, hardened layer that cracks prematurely under tension; cracks then propagate inward, causing fracture before the interior can fully deform. UV aging is volumetric: deeper penetration triggers uniform photo-oxidative crosslinking throughout the material, restricting chain mobility and causing progressive embrittlement; cracks thus propagate rapidly across the entire cross-section because strain energy cannot be dissipated by localized chain slippage and rearrangement, leading to a more severe loss of ductility.

#### 3.1.4. Patterns of Change in the Modulus of Elasticity

**Figure 6:**
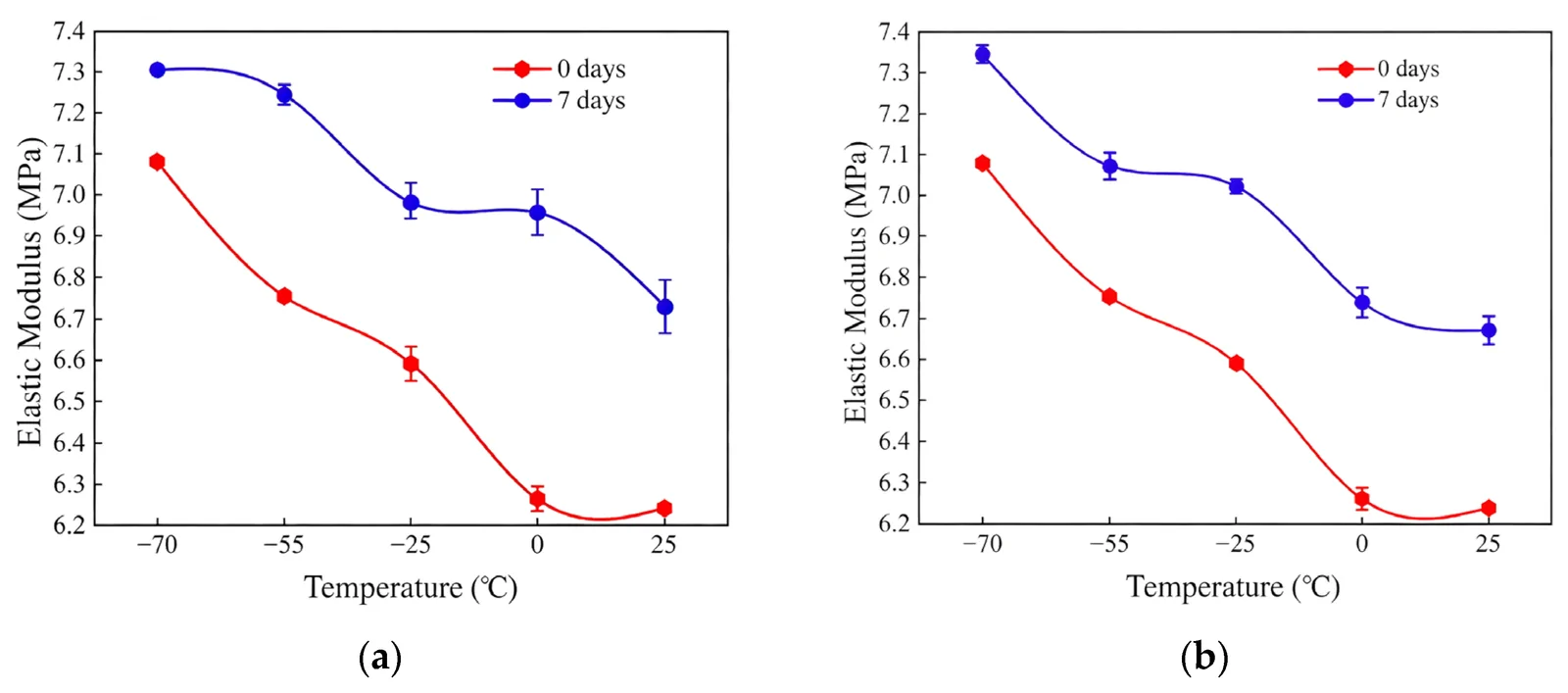
Elastic modulus trends under different aging conditions. (**a**) Elastic modulus change patterns under ozone aging. (**b**) Elastic modulus change patterns under UV aging.

In both aging environments, the elastic modulus of phenyl silicone rubber gradually decreased with increasing temperature but was higher at each temperature than that of the unaged material. As shown in Figure 6, ozone aging raised the modulus by 0.2–0.7 MPa, and UV aging by 0.3–0.5 MPa, indicating that both environments caused irreversible stiffening, though to slightly different extents.

As shown in Figure 6, ozone aging raised the elastic modulus by 0.2–0.7 MPa, while UV aging raised it by 0.3–0.5 MPa, indicating comparable overall stiffening. However, their temperature dependencies differed: UV produced a relatively stable increase due to uniform photo-oxidative crosslinking throughout the bulk, whereas ozone-induced variation was larger (0.2–0.7 MPa) because its surface-limited attack created a crosslinking gradient, making the modulus more sensitive to temperature.

### 3.2. Aging Properties of Phenyl Silicone Rubber

#### 3.2.1. Compression Set

**Figure 7:**
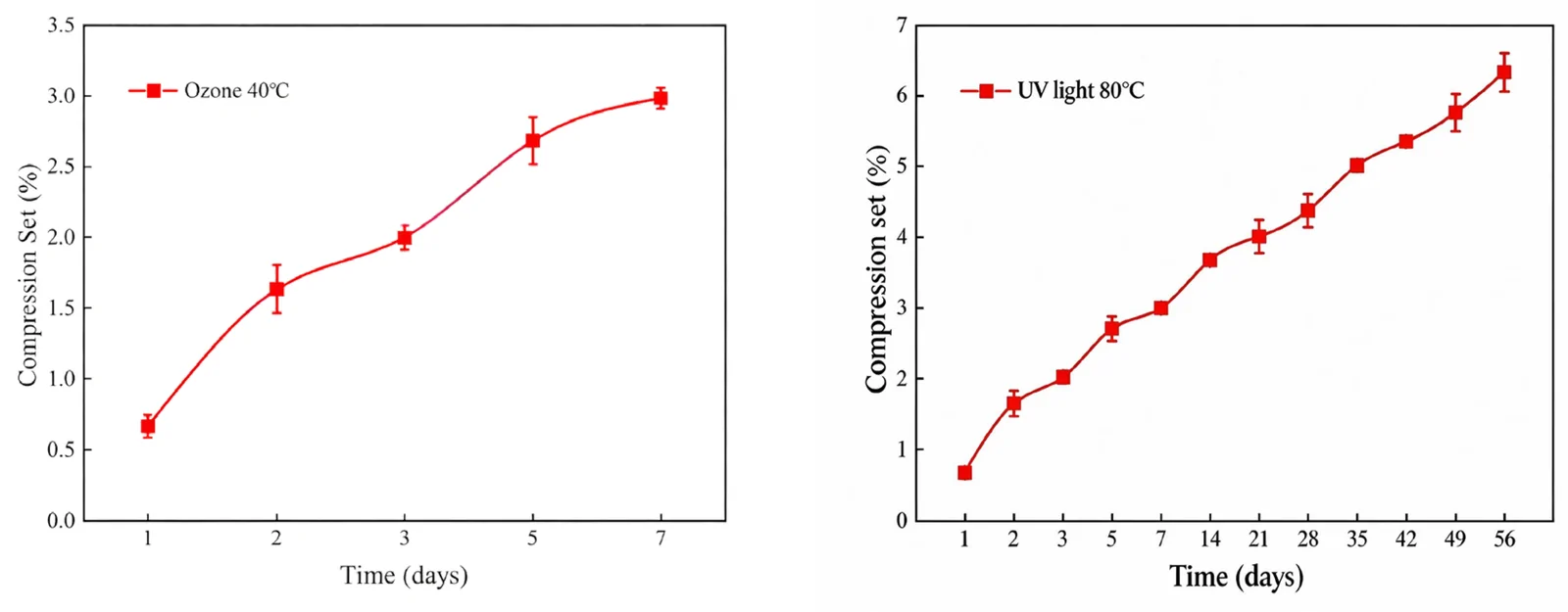
Compression set of phenyl silicone rubber under various aging conditions.

As shown in Figure 7, the compression set of cylindrical phenyl silicone rubber specimens under ozone exposure increased steadily with aging time. At 1 day, it was 0.67%, with an intact crosslinked network, ample chain mobility, and good elastic recovery. With prolonged aging, the combined action of ozone and the 40 °C environment drove a gradual rise, reaching 2.97% at 7 days, because increasing crosslink density restricted chain movement and prevented full elastic recovery after compressive deformation. Under UV exposure, the compression set of the specimens increased steadily with aging time. At 1 day it was 0.68%, with an intact crosslinked network, ample chain mobility, and good elastic recovery. With continued aging, the combined effects of sustained UV radiation and the 80 °C environment led to a gradual rise, reaching 3.00% at 7 days. This rapid increase shows that UV and high temperature synergistically trigger photodegradation and crosslinking restructuring, densifying the network and restricting chain movement, thereby reducing elastic recovery. After 7 days, the compression set continued to rise in a nearly linear manner, reaching 3.679% at 14 days, 5.763% at 49 days, and 6.333% at 56 days, with only a slight slowing of the rate. This steady accumulation indicates that the synergistic effect of UV and high temperature persisted and intensified: high-energy UV radiation broke molecular chains, while the 80 °C environment accelerated chain motion and radical diffusion, promoting secondary crosslinking that further densified the network and severely restricted chain mobility. As a result, the material could not recover elastically after compressive deformation, leading to a continuous loss of recovery and steady accumulation of compression set.

#### 3.2.2. Surface Topography Evolution

**Figure 8:**
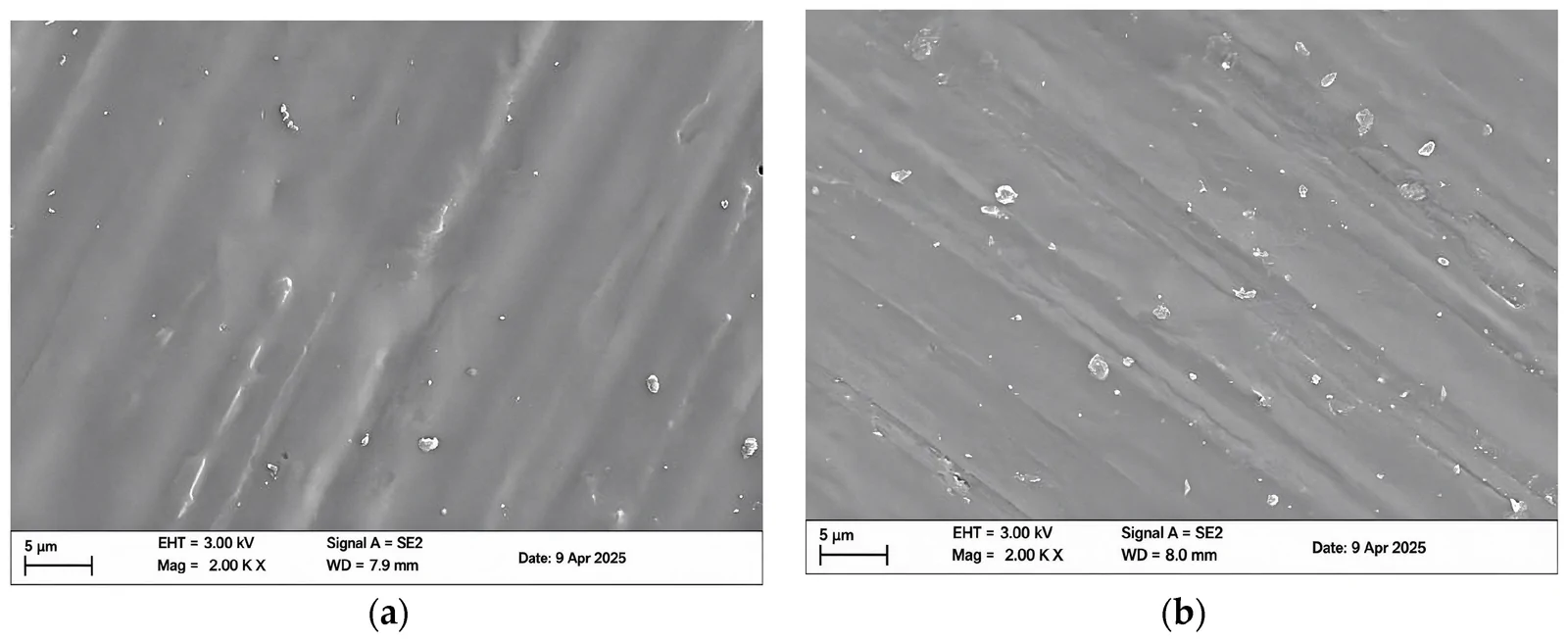
Surface morphology of cylindrical specimens after ozone aging. (**a**) Surface morphology before aging. (**b**) Surface morphology after 7 days of ozone aging.

As shown in Figure 8, 7-day ozone aging markedly altered the surface morphology: the unaged surface displayed a uniform rough texture with a continuous, intact structure and no visible defects, while the aged surface retained the original texture but showed numerous irregularly distributed microscopic particles with slight local agglomeration. These changes stem from ozone’s strong oxidative attack on unsaturated bonds, causing localized chain scission; the resulting reactive fragments, driven by the 40 °C thermal environment, undergo simultaneous re-crosslinking and further breakage. Some broken chains and oxidation-generated small molecules fail to re-crosslink and instead deposit on the surface, aggregating into the fine particles observed under SEM.

The formation of surface particles not only alters the morphology but also degrades macroscopic mechanical behavior. Particle-rich regions introduce numerous microdefects and stress concentrations in the surface layer, promoting preferential crack initiation under tension, which corroborates the earlier decrease in elongation at break. Together with the surface hardening layer, these particles prevent the material from achieving its full ductility, as premature microcrack initiation and propagation lead to earlier macroscopic fracture.

**Figure 9:**
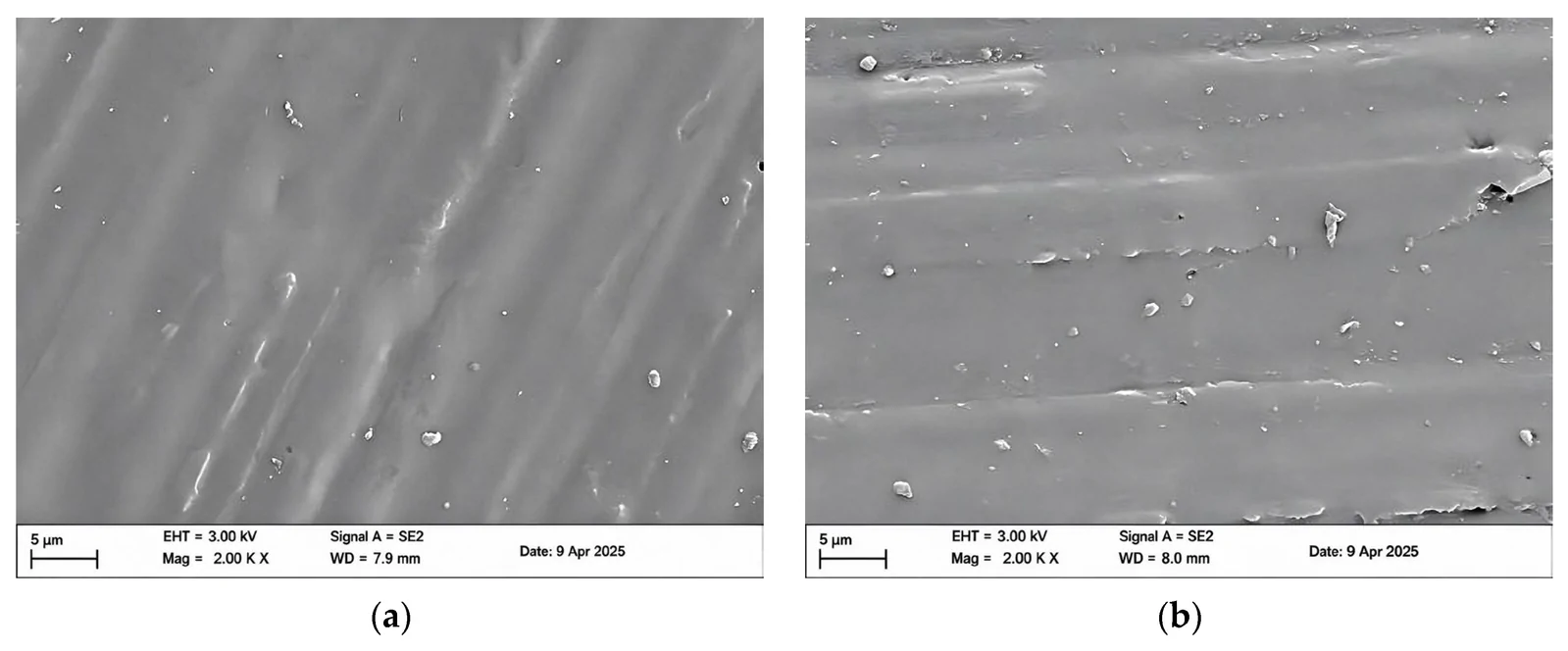

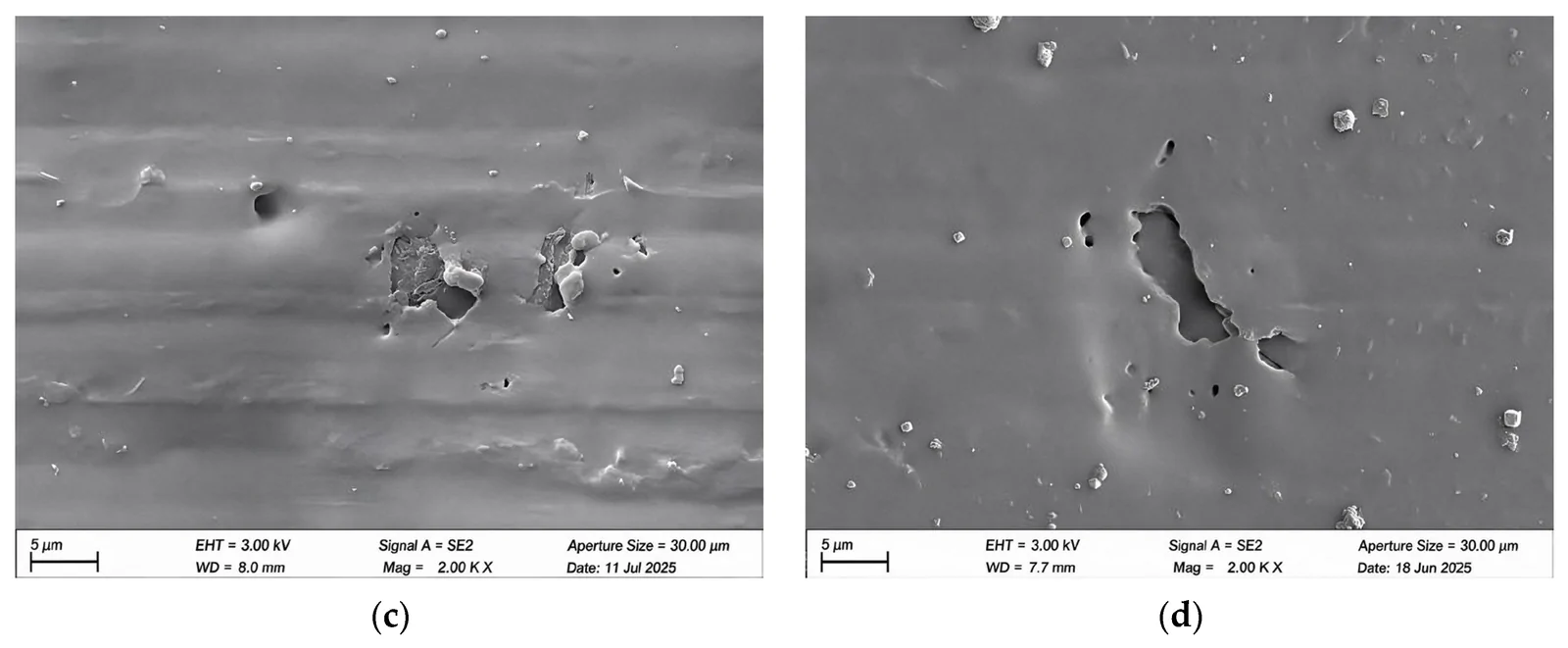
Surface morphology of cylindrical specimens after UV aging. (**a**) Surface morphology before aging. (**b**) Surface morphology after 7 days of UV aging. (**c**) Surface morphology after 28 days of UV aging. (**d**) Surface morphology after 56 days of UV aging.

Under UV exposure, the surface microstructure of phenyl silicone rubber evolved in distinct stages from slight degradation to severe damage, with both the extent and rate of degradation far exceeding those in ozone. As shown in Figure 9, after 7 days the original uniform rough texture remained recognizable, but numerous microscopic particles had precipitated. This resulted from the synergistic effect of high-energy UV photons, which directly break molecular bonds and trigger photodegradation, and the 80 °C environment, which accelerates radical diffusion and crosslinking restructuring; chain fragments and oxidation products, unable to be removed, deposited and aggregated into fine particulates. By 28 days, deterioration accelerated: the uniform texture was locally disrupted, damaged areas appeared, and irregular small pores and debris accumulations formed. Continued UV and thermal exposure drove progressive chain scission and crosslinking, raising surface crosslink density; excessively crosslinked brittle microzones fractured under internal thermal and residual stresses, with detached debris leaving pores, while stress concentrations from uneven crosslinking propagated defects from original particle-rich regions toward the surface, significantly eroding morphological integrity. After 56 days, the surface suffered severe damage: the original texture nearly disappeared, replaced by large irregular pits with rough edges and secondary micropores, with few scattered particles remaining. This end-stage morphology arose from the long-term synergistic evolution of chain scission, void accumulation, and inhomogeneous crosslinking—continuous internal chain breakage generated microvoids, while the rigid over-crosslinked network prevented their closure and repair; under repeated thermal and environmental stresses, microvoids expanded and coalesced into macroscale pits, fundamentally compromising surface structural integrity.

From a microstructural evolution perspective, UV aging causes distinctly cumulative and irreversible damage to phenyl silicone rubber. The 7-to-28-day period is a critical window in which the surface morphology shifts from slight degradation to severe damage, and by 56 days the original continuous structure is completely lost. This loss of surface integrity closely correlates with the sustained decline in mechanical properties—pits and voids act as macroscopic crack initiation sites that significantly reduce the load-bearing capacity and sealing reliability of seals in service.

#### 3.2.3. Infrared Analysis of Molecular Structure Changes

**Figure 10:**
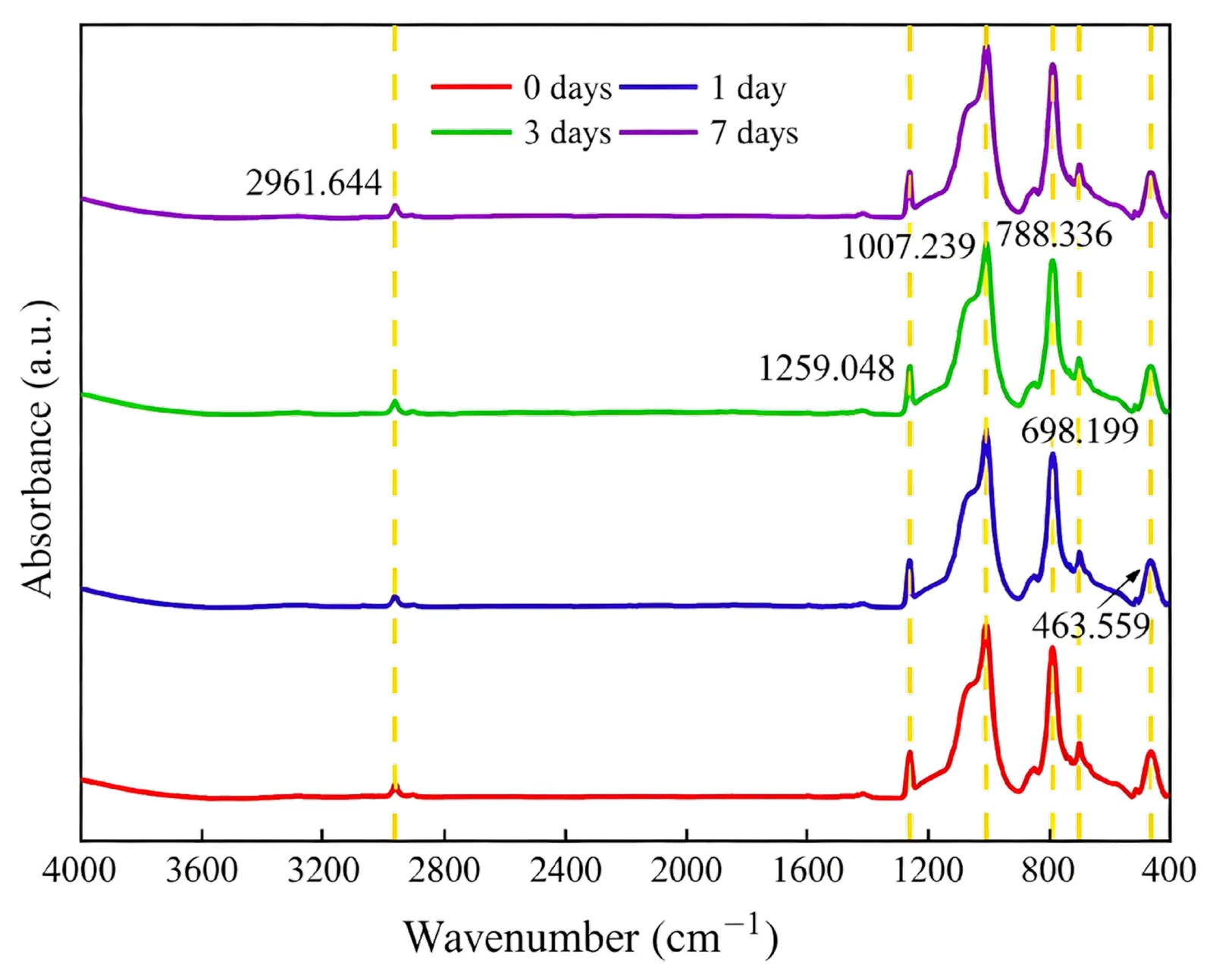
Infrared spectra of ozone-aged phenyl silicone rubber.

Figure 10 shows the infrared spectra (4000–400 cm^−1^) of phenyl silicone rubber during ozone aging. Spectral analysis reveals an overall pattern of stable side chains, cross-linked main chains, and an intact backbone. The C-H absorption of Si-CH_3_ at 2961.644 cm^−1^ remained essentially unchanged, indicating that methyl side groups are inert to ozone. The peaks at 1259.048 cm^−1^ (Si-O-Si) and 1007.259 cm^−1^ (Si-OH/Si-O-Si) increased slightly, confirming that ozone oxidation drives a dynamic process of main-chain scission and re-crosslinking via silanol condensation, which raises the crosslink density. Meanwhile, the peaks for Si-CH_3_ at 788.336 cm^−1^, the benzene ring at 698.199 cm^−1^, and Si-O-Si at 463.559 cm^−1^ remained nearly unchanged, indicating that the phenyl side groups and the siloxane backbone were not substantially oxidized [38,39,40]. No characteristic peaks shifted or split after aging, confirming the excellent ozone resistance of the phenyl silicone rubber backbone. Chemically, aging primarily involves moderate crosslink-network restructuring rather than a fundamental change in molecular structure. This molecular-level evolution forms a clear causal chain with the macroscopic trends: hardness and elastic modulus increase, while tensile strength and elongation at break decrease.

**Figure 11:**
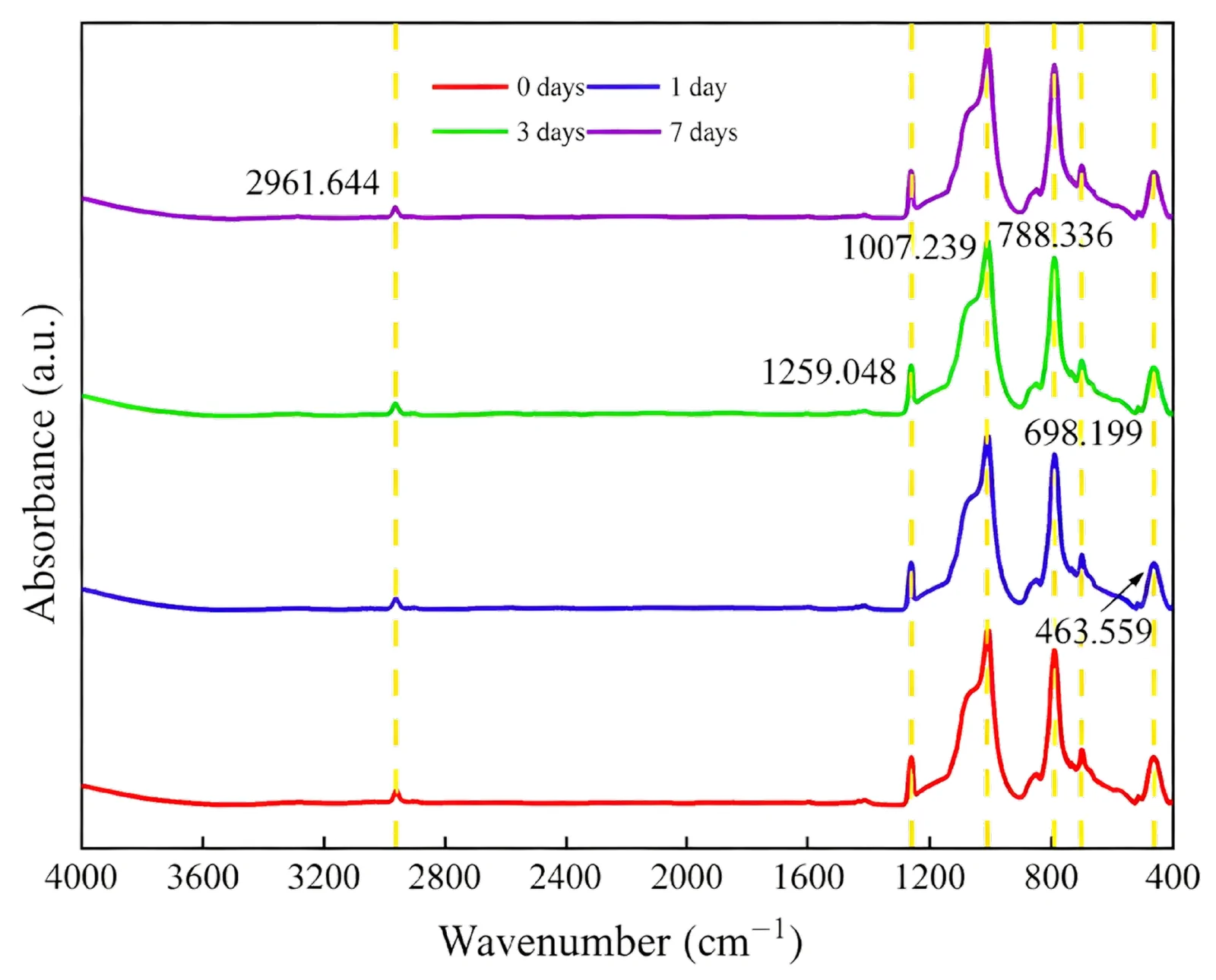
Infrared spectra of UV-aged phenyl silicone rubber.

Figure 11 reveals the chemical mechanisms and property evolution of phenyl silicone rubber under UV aging, showing a multilevel response—side-chain reactions, enhanced main-chain crosslinking, and overall backbone stability—distinct from ozone aging. The slight enhancement of the Si-CH_3_ C-H peak at 2961.644 cm^−1^ indicates that high-energy UV triggers radical oxidation and restructuring of methyl side chains, sharply contrasting with their ozone inertness and reflecting UV’s stronger chemical activation. The marked intensification of Si-O-Si peaks at 1259.048 cm^−1^ and 1007.259 cm^−1^ confirms intensified photo-oxidative crosslinking of the main chain via silanol condensation, significantly raising crosslink density. Meanwhile, the Si-CH_3_ (788.336 cm^−1^), benzene ring (698.199 cm^−1^), and Si-O-Si (463.559 cm^−1^) peaks remained essentially unchanged, indicating no fundamental degradation of phenyl side groups or the siloxane backbone. No characteristic peaks shifted or split, confirming good backbone weather resistance under UV; however, the methyl side-chain changes demonstrate that UV aging has broader chemical effects, strengthening the main-chain crosslinking network and triggering side-chain rearrangements. This molecular-level evolution provides direct chemical-mechanism support for the more severe mechanical deterioration caused by UV aging.

### 3.3. Effect of Aging on Contact Pressure

**Figure 12:**
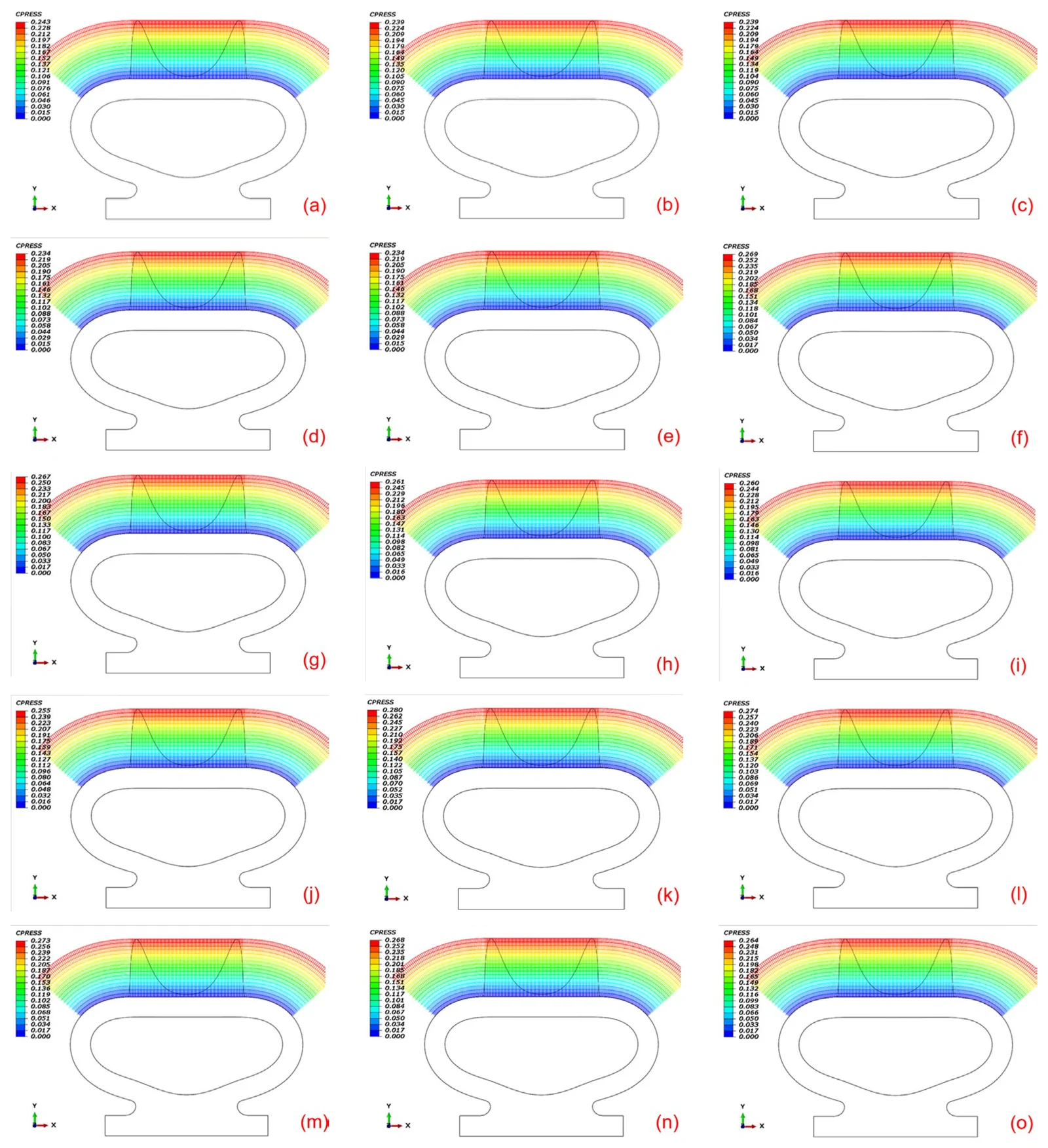
Contact pressure distribution of the Omega-profile seal across temperatures (−70 °C, −55 °C,−25 °C, 0 °C and 25 °C) under different states: (**a**–**e**) Unaged conditions; (**f**–**j**) UV aging conditions; (**k**–**o**) Ozone aging conditions.

Figure 12 presents the contact pressure contours for an Omega-type seal at five temperatures (−70 °C, −55 °C, −25 °C, 0 °C, and 25 °C) under unaged, 7-day ozone-aged, and 7-day UV-aged conditions. At −70 °C, the unaged contact pressure is concentrated in the sealing lip region, with a maximum contact pressure of 0.243 MPa. After ozone aging, the maximum increases to 0.280 MPa; after UV aging, it reaches 0.269 MPa, which is higher than the unaged value but slightly lower than the ozone-aged value. In all three conditions, the maximum contact pressure decreases with increasing temperature. These results indicate that both aging environments increase the contact pressure at the sealing interface, confirming that aging-induced material hardening enhances the contact characteristics.

**Figure 13:**
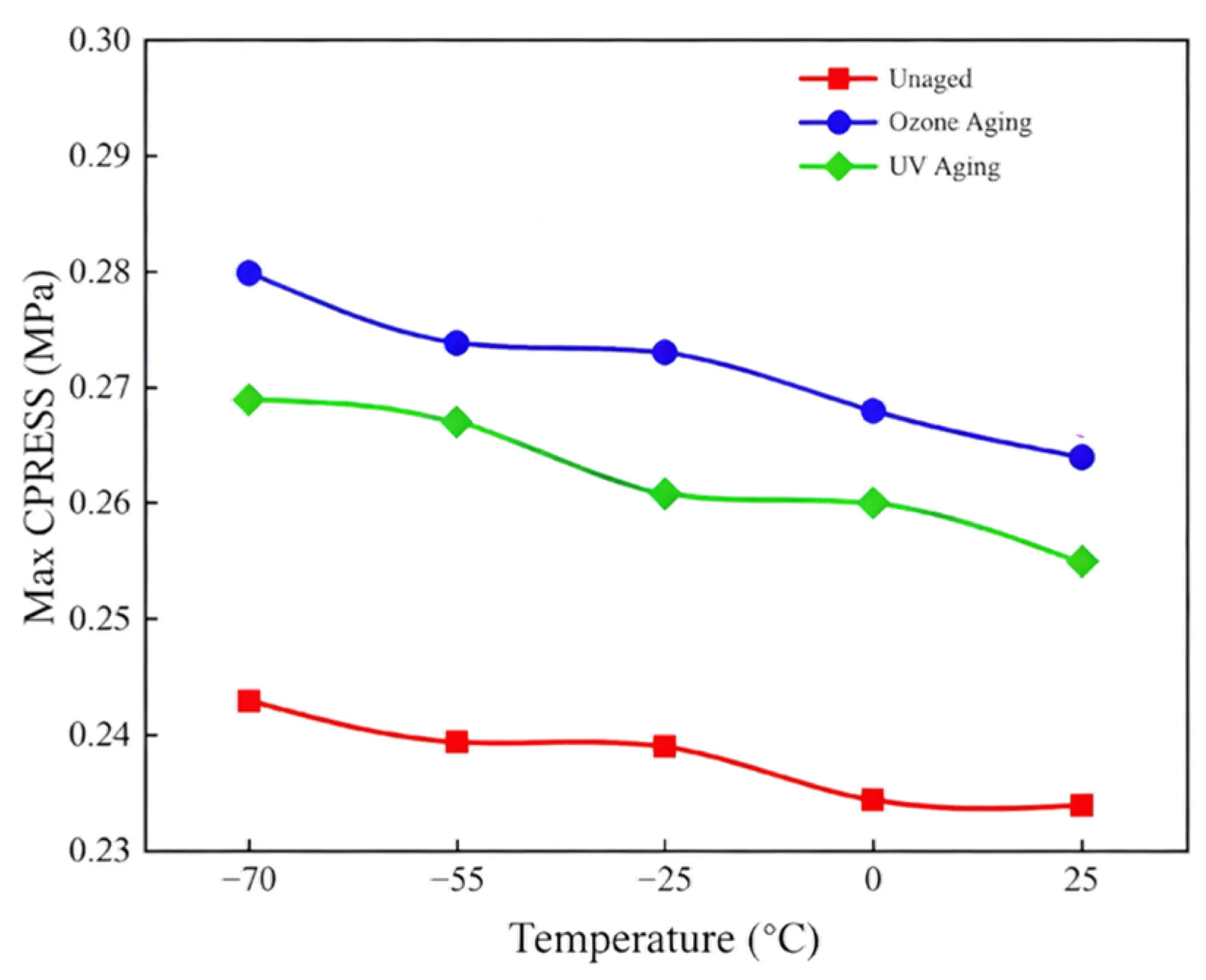
Variation in maximum contact stress of the Omega-type seal under different aging conditions.

Figure 13 compares the maximum contact pressure of the Omega-type seal under unaged, ozone-aged, and UV-aged conditions from −70 °C to 25 °C. In all three states, the maximum contact pressure decreased gradually with rising temperature, declining by 0.016 MPa, 0.014 MPa, and 0.009 MPa for the ozone-aged, UV-aged, and unaged specimens, respectively. This trend reflects the temperature dependence of the elastic modulus of phenyl silicone rubber: at low temperatures, restricted molecular chain motion stiffens the material while the seal remains under a preset compressed displacement with a nearly constant contact area (14.0078 mm^2^), thereby increasing normal contact forces and maximum contact pressure. As the temperature rises toward room temperature, molecular chain motion increases, the elastic modulus decreases, and the maximum contact pressure correspondingly declines [41].

At each temperature, the maximum contact pressure followed the order ozone-aged > UV-aged > unaged. Compared with the unaged specimens, the increases were approximately 15.2% and 10.7% at −70 °C, 14.6% and 11.7% at −55 °C, 14.2% and 9.2% at −25 °C, 14.5% and 11.1% at 0 °C, and 12.8% and 9.0% at 25 °C for the ozone- and UV-aged specimens, respectively. These differences arise because ozone and UV aging accelerate molecular chain scission and re-crosslinking, amplifying the stiffening effect and increasing the elastic modulus, which ultimately raises the maximum contact pressure at all temperatures.

## 4. Conclusions

By combining macroscopic mechanical testing, microstructural characterization, and finite element simulation, this study systematically elucidated the aging mechanical behavior and underlying mechanisms of phenyl silicone rubber in two high-altitude environments: ozone and ultraviolet (UV) radiation. Both environments restructured the cross-linked network, increasing stiffness and reducing ductility, but UV aging caused more severe degradation—the hardness increase (2.30 HA) was about 1.7 times that of ozone aging (1.35 HA), and the maximum loss in elongation at break reached 29%, versus 16% for ozone. Microstructural analysis showed that UV aging follows a complete failure sequence of particle precipitation, localized damage, and through-penetrating pits; infrared spectroscopy further confirmed that UV strengthens main-chain cross-linking and actively restructures methyl side chains, yielding a much broader chemical impact than ozone. The compression set rose to 6.33% after 56 days of UV aging and increased almost linearly, indicating a progressive loss of elastic compensation capacity during long-term service—a cumulative effect more threatening than short-term mechanical degradation. Finite element simulation showed that ozone and UV aging increased contact pressure by approximately 15.2% and 10.7%, respectively. Thus, UV radiation should be regarded as the dominant aging factor in high-altitude seal design, with priority given to UV-resistant formulations or surface protection and to accurate assessment of long-term sealing reliability.

## Figures and Tables

**Table 1:** Schedule for removing test specimens from the ozone environmental aging test.

Test Specimen Types	Aging Time (Days)				
Dumbbell-shaped	—	—	—	—	7
Cylindrical	1	2	3	5	7

**Table 2:** Schedule for removing test specimens from the UV environmental aging test.

Test Specimen Types	Aging Time (Days)											
Dumbbell-shaped	—	—	—	—	7	—	—	—	—	—	—	—
Cylindrical	1	2	3	5	7	14	21	28	35	42	49	56

## Data Availability

The original contributions presented in this study are included in the article. Further inquiries can be directed to the corresponding author.

## References

[1] Mishev A.L., Kodaira S., Kitamura H., Ploc O., Ambrožová I., Tolochek R.V., Kartsev I.S., Shurshakov V.A., Artamonov A.A., Inozemtsev K.O. (2023). Radiation environment in high-altitude Antarctic plateau: Recent measurements and model studies. Sci. Total Environ..

[2] Semple J., Moore G. (2020). High levels of ambient ozone (O3) may impact COVID-19 in high altitude mountain environments. Respir. Physiol. Neurobiol..

[3] Chien A., Maxwell R., Chambers D., Balazs B., LeMay J. (2000). Characterization of radiation-induced aging in silica-reinforced polysiloxane composites. Radiat. Phys. Chem..

[4] Zhou M.Z., Wang H.R., Guo X., Wei Y.C., Liao S. (2023). Synergistic effect of thermal oxygen and UV aging on natural rubber. e-Polymers.

[5] Zheng T., Zheng X., Zhan S., Zhou J., Liao S. (2021). Study on the ozone aging mechanism of Natural Rubber. Polym. Degrad. Stab..

[6] Liang M., Fu Z., Guo M., Du X., Tan Y. (2024). Effect of ozone aging on the rheological and chemical properties of crumb rubber-modified bitumen. Road Mater. Pavement Des..

[7] Wang Q., Liu H., Li Y., Li W., Sun D., Zhao H., Tie C., Gu J., Zhao Q. (2024). Predicting plateau atmospheric ozone concentrations by a machine learning approach: A case study of a typical city on the southwestern plateau of China. Environ. Pollut..

[8] Rumiana B., Plamen M., Nikolay M. (2022). Dependence of the Index of Biologically Active Ultraviolet Radiation on the Season and Time of Day. Atmosphere.

[9] Blumthaler M., Ambach W., Ellinger R. (1997). Increase in solar UV radiation with altitude. J. Photochem. Photobiol. B Biol..

[10] Wang C., Shi G., Du L., Ni X., Hu Y., Pang D., Meng J. (2024). The source of volatile organic compounds pollution and its effect on ozone in high-altitude areas. Ecotoxicol. Environ. Saf..

[11] Ning K., Xie P., Fu Z., Wang B., Jiang Z., Tan Z., Liao Z., Jiang L. (2025). Research on Ultraviolet Resistance Method of Composite Insulators Silicone Rubber Doped with Nano-TiO_2_. J. Appl. Polym. Sci..

[12] Liu J., Fang Z., Bao C. (2024). Studies on 8.4 W/m·K thermally conductive silicone rubber with high compressibility, high electrical insulation, high thermal reliability, and low cost. Compos. Part A.

[13] Zhang Z., Zhang Z., Yue S., Jiang X., Hu J. (2023). Performance Characteristics of Silicone Rubber for Use in Acidic Environments. Polymers.

[14] Huang Z., Chen Y. (2026). An Overview of Silicone Rubber: From Properties to Industry. Chin. J. Chem..

[15] Zhu Q., Wang Z., Zeng H., Yang T., Wang X. (2021). Effects of graphene on various properties and applications of silicone rubber and silicone resin. Compos. Part A.

[16] Cui T., Lin C.-W., Chien C., Chao Y., Van Zee J. (2011). Service life estimation of liquid silicone rubber seals in polymer electrolyte membrane fuel cell environment. J. Power Sources.

[17] Wang Y., Cao R., Wang M., Liu X., Zhao X., Lu Y., Feng A., Zhang L. (2020). Design and synthesis of phenyl silicone rubber with functional epoxy groups through anionic copolymerization and subsequent epoxidation. Polymer.

[18] Lu Z., Tian Y., Xing Y., Luo Y., Zou H., Zhou S. (2025). Constructing layered structure improves thermal protection performance of silicone rubber-based composites under coupled mechanical-thermal-oxidative conditions. Constr. Build. Mater..

[19] Yu F., Lu A., Lu J., Wang Z., Zhang Q., Geng C., Li Z. (2019). Effect of phenyl content, sample thickness and compression on damping performances of silicone rubber: A study by dynamic mechanical analysis and impact damping test. Polym. Test..

[20] Guo H., Yang J., Deng W., Li X., Nie Y., Zhu Y., Li G., Wei Y. (2024). Effect and mechanism of phenyl content on the electrical insulation properties of silicone rubber. J. Appl. Polym. Sci..

[21] Maiti A., Small W., Kroonblawd M.P., Lewicki J.P., Goldman N., Wilson T.S., Saab A.P. (2021). Constitutive Model of Radiation Aging Effects in Filled Silicone Elastomers under Strain. J. Phys. Chem. B.

[22] Kim J., Kim Y., Cho Y. (2025). Estimation of Synthetic Rubber Lifespan Based on Ozone Accelerated Aging Tests. Polymers.

[23] Li S., Ke Y., Xie L., Zhao Z., Huang X., Wang Y., Wang Z. (2023). Study on the aging of three typical rubber materials under high- and low-temperature cyclic environment. e-Polymers.

[24] Han R., Wang Z., Zhang Y., Niu K. (2019). Thermal stability of CeO2/graphene/phenyl silicone rubber composites. Polym. Test..

[25] Han R., Quan X., Shao Y., Niu K. (2020). Tribological properties of phenyl-silicone rubber composites with nano-CeO2 and graphene under thermal-oxidative aging. Appl. Nanosci..

[26] Han X., Zhao K., Hao J., Wang H., Zhu L., Zhou C. (2025). High-performance silicone rubber composite via interface effect: Surpassing the damping property-mechanical strength-aging resistance trade-off in extreme environments. J. Mater. Res. Technol..

[27] (2025). Rubber—General Procedures for Preparing and Conditioning Test Pieces for Physical Test Methods.

[28] Xu C., Lin J., Kong H., Jin J., Chen L., Xu X. (2025). Rapid increases in ozone concentrations over the Tibetan Plateau caused by local and non-local factors. Atmos. Chem. Phys..

[29] Maghrabi A.H., Aldosari A.F. (2025). The Impact of Space Weather on Ultraviolet Radiation at Mid Latitudes. Adv. Meteorol..

[30] Liu Q., Hou F., Ge D., Lv S., Ju Z. (2025). Study on Performance and Aging Mechanism of Rubber-Modified Asphalt Under Variable-Intensity UV Aging. Materials.

[31] Shi H., Wu J., Chen Z., Cao P., Zhou T., Su B., Wang Y. (2026). Sealing performance of phenyl-silicone rubber based on constitutive model under thermo-oxidative aging. Polymers.

[32] Jiao X., Yu J., Yin B., Sun H. (2025). Effect of Thermo-Oxidative, Ultraviolet, and Ozone Aging on Nonlinear Viscoelasticity of Carbon Black-Filled Rubber Based on Crosslink Density Evolution. Polym. Compos..

[33] Zhou J., Deng P., Gui H., Xu J., Liao J., Ding A. (2023). Enhanced Ozone Aging Resistance of Natural Rubber with 2-Mercaptobenzothiazole as the Constant-Viscosity Agent. J. Chem..

[34] Zhao M., Zhao C., Han C., Wu Y. (2024). Ozone resistance of three natural antioxidants in solution polymerized styrene-butadiene rubber—Molecular simulation and experimental study. Polym. Degrad. Stab..

[35] Dou R., Zhang Y., Huang Z., Liu Q., Huang W., Meng X., Chen H. (2024). Study on the aging behavior and mechanism of nitrile rubber composites in combined radiation-thermal environments. J. Anal. Appl. Pyrolysis.

[36] Wang R.Y., Dou Z.F., Liu Z.H., Li N., Liu X.R., Zhang W.F. (2025). Research on ultraviolet degradation behavior and aging mechanisms of fluorosilicone rubber in simulated tropical marine atmospheric environment. Polym. Degrad. Stab..

[37] Jin F., Zhou Y., Liang B., Liang X., Zhou Z., Zhang L. (2019). Effect of UV radiation aging on creepage discharge characteristics of high temperature vulcanized silicon rubber at high altitude. Plasma Sci. Technol..

[38] Zhang Z., Liang T., Jiang Z., Jiang X., Hu J., Pang G. (2022). Application of Infrared Spectroscopy in Research on Aging of Silicone Rubber in Harsh Environment. Polymers.

[39] Zhang K., Zhang C., Zhou Z., Liu Z., Deng Y., Gu C., Zhou S., Sun D., Liu H., Yu X. (2025). An Ensemble Learning Model for Aging Assessment of Silicone Rubber Considering Multifunctional Group Comprehensive Analysis. Polymers.

[40] Yu X., Zhang K., Zhou Z., Liu Z., Deng Y., Gu C., Zhou S., Sun D. (2026). Integrated Learning-Based Ageing Assessment of Silicone Rubber. High Volt..

[41] Wei Q., Wu J., Chen Z., Su B., Wang Y. (2025). Evolution of tribological mechanisms and interfacial contact behavior of nitrile butadiene rubber under thermal–oxidative aging. Tribol. Int..

